# Pioglitazone-Based Combination Approaches for Non-Small-Cell Lung Cancer

**DOI:** 10.3390/pharmaceutics17111416

**Published:** 2025-10-31

**Authors:** Sravya Aluru, Anita Thyagarajan, Ravi P. Sahu

**Affiliations:** Department of Pharmacology and Toxicology, Boonshoft School of Medicine at Wright State University, Dayton, OH 45435, USA; aluru.6@wright.edu (S.A.); anita.thyagarajan@wright.edu (A.T.)

**Keywords:** non-small-cell lung cancer, peroxisome proliferator-activated receptor-gamma, pioglitazone, cell signaling pathways, cancer therapy

## Abstract

Lung cancer remains the leading cause of cancer-related mortality worldwide, with non-small-cell lung cancer (NSCLC) being the most prevalent subtype. NSCLC is marked by a complex genetic makeup, involving numerous driver mutations and epigenetic changes that drive tumor growth and resistance to treatment. While several approaches, including chemotherapy and targeted therapy, have been used for lung cancer treatment, their overall responses remain dismal, indicating the need to explore alternative targets implicated in cancer growth. Among various candidates, peroxisome proliferator-activated receptor-gamma (PPARγ), which plays critical roles in regulating cellular functions related to tumorigenesis, has been explored as a promising target for NSCLC intervention. To that end, thiazolidinediones, including pioglitazone, that target PPARγ have shown promise in multiple cellular and preclinical models of NSCLC. Mechanistically, pioglitazone inhibits cancer growth and induces apoptosis via downregulating key signaling pathways, including mitogen-activated protein kinase (MAPK), which play critical roles in regulating cellular activities such as epithelial-to-mesenchymal transition (EMT), cellular bioenergetics, and glucose metabolism. This review highlights the recent updates on the mechanistic insights and the efficacy of PPARγ agonist-based approaches, with an emphasis on pioglitazone, for the treatment of NSCLC. We logically discuss the experimental evidence from the in vitro and in vivo studies exploring pioglitazone’s effect on metabolic pathways, chemical-carcinogen-induced tumorigenesis, the targeting of cell signaling pathways, and then its combination with other therapeutic agents. We also present clinical studies that support pioglitazone’s potential in chemoprevention and underscore its further exploration in large cohorts of NSCLC patients.

## 1. Introduction

According to the 2025 American Cancer Society, lung cancer has been estimated to be the second most common cancer and the leading cause of cancer deaths [1]. While lung cancer cases have been in a steady decline, with an annual decrease of 2.5% in men and 1% in women, the mortality rates have been increasing, with higher reported cases in males than in females [2,3]. Based on histology, lung cancer is divided into two major subtypes, where non-small-cell lung cancer (NSCLC) is the most common subtype, accounting for 85% of all cases, and the second, less prevalent, subtype is small-cell lung cancer (SCLC) [4,5,6]. NSCLC is further categorized into three major subtypes, adenocarcinoma, squamous cell carcinoma, and large cell carcinoma, along with less common subtypes, including adenosquamous carcinoma and sarcomatoid carcinoma [7,8,9]. NSCLC’s risk factors are generally classified into two major groups: the modifiable risk factors include tobacco smoking, exposures to radon, asbestos, arsenic, and environmental contaminants such as biomass fuels and industrial carcinogens, while unmodifiable risk factors include air pollution and a personal or family history of lung cancer [10,11,12,13].

The treatment approach for lung cancer depends on the tumor stage, histology, genetic alterations, and the patient’s condition, which includes surgery, radiotherapy, chemotherapy, immunotherapy, and targeted therapy [14,15,16]. Notably, targeted therapy is being used to manage advanced forms of cancer, particularly NSCLC. While no substantial benefits in the overall survival in early-stage NSCLC were noted, notable improvements in clinical outcomes were documented with targeted therapy in patients with advanced disease [14]. Nevertheless, the majority of lung cancer patients develop drug resistance to targeted therapy, indicating the need for the implementation of novel approaches, including considering those potential factors that regulate tumor growth. To that end, recent studies have highlighted the role of peroxisome proliferator-activated receptors (PPARs) in regulating cellular functions related to tumorigenesis, with PPAR gamma (PPARγ) being a promising isoform [17]. PPARγ, a nuclear receptor superfamily 1 group C member 3 (NR1C3), is a ligand-activated transcription factor that controls the expression of genes related to lipid and glucose metabolism, energy homeostasis, cell differentiation, and inflammation [11,18,19,20]. While NSCLC cells express high levels of PPARγ, their natural activators, such as 15-deoxy-Δ12,14 prostaglandin J2, are not strong enough to maximize their anticancer potential. Therefore, using an external activator of PPARγ could help in NSCLC treatment [21]. Thiazolidines (TZD) are a class of heterocyclic compounds that include pioglitazone, rosiglitazone, and troglitazone that primarily act by targeting PPARγ [22,23,24,25]. Among these PPARγ agonists, pioglitazone is commonly used for the treatment of T2DM and exhibits anticancer properties, including inhibiting cancer cell proliferation, invasion, migration, and resistance to apoptosis, which are mediated by downregulating certain genes that interfere with carcinogenic cell pathways [11,21,26,27].

This review provides an in-depth exploration of pioglitazone, specifically its mechanisms of action and efficacy as a monotherapy or in combination with other neoplastic agents in the treatment of NSCLC. We logically discuss the experimental evidence from the in vitro and in vivo studies that support pioglitazone’s therapeutic potential and its effectiveness in lung cancer treatment. Additionally, the review discusses the diverse mechanisms through which PPARγ activation influences various cellular processes, such as apoptosis, cell cycle regulation, metastasis, and immune modulation, ultimately exerting anticancer effects in NSCLC. Through this analysis, we aim to highlight the promising efficacy of pioglitazone for NSCLC.

## 2. Mechanisms of Action of PPARγ Agonists

**Figure 1:**
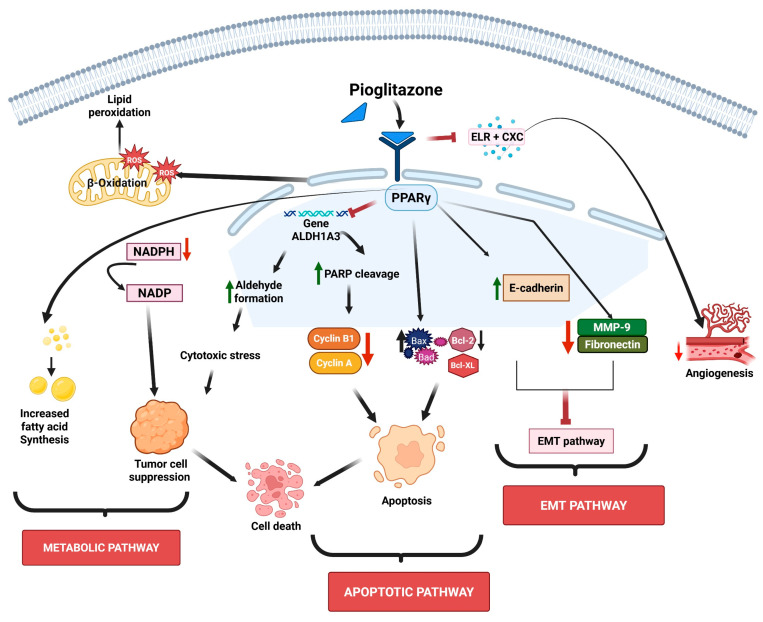
Schematic representation of pioglitazone’s mechanisms of action. Pioglitazone targets multiple cellular properties such as lipid peroxidation, aldehyde formation, and chemokines and signaling cascades, leading to alterations in the metabolic pathway, inhibition of EMT and angiogenesis, and induction of apoptosis. The sign green arrow denotes downregulation, red arrow denotes upregulation or increased expression, and ┴ denotes inhibition.

Metabolic reprogramming plays a key role in supporting tumor growth. Therefore, targeting lipid metabolism has emerged as a promising anticancer strategy [28,29]. PPARγ plays a central regulatory role in enhancing fatty acid synthesis and β-oxidation in lung cancer, and pioglitazone induces PPARγ activation and promotes de novo fatty acid synthesis and β-oxidation. In addition, depletion of nicotinamide adenine dinucleotide phosphate (NADPH) and disruption of redox balance by pioglitazone also mediate tumor growth suppression [29]. The schematic representation of pioglitazone’s mechanisms of action is shown in Figure 1.

Troglitazone, another PPARγ agonist, has also been shown to promote a metabolic shift in lung alveolar carcinoma cells, thereby enhancing fatty acid oxidation as the primary energy source [30]. As aldehyde dehydrogenases (ALDHs) scavenge aldehydes during lipid peroxidation and are highly active in lung cancer [31], increased expression of ALDH1A1 and ALDH3A1 has been noted in squamous cell carcinomas and adenocarcinomas of the lung, as well as in atypical pneumocytes of nonsmokers and in normal pneumocytes of smokers [32,33]. Thus, the inhibition of ALDHs exposes cells to toxic aldehydes, which results in the induction of apoptosis [34]. Notably, PPARγ downregulates ALDHs to suppress lung cancer growth, as seen with arachidonic acid-induced PPARγ activation, which reduces ALDH3A1 and increases lipid peroxidation in A549 cells [35]. TZD-mediated PPARγ activation also inhibits ALDH1A3 expression, exerting anti-proliferative effects in H1993 cells [11].

As apoptosis helps maintain healthy cell populations in normal tissues, tumor cells often evade this process to keep growing uncontrollably [36,37]. Apoptosis can occur through two main pathways: the intrinsic and the extrinsic pathways. Though these pathways have different stimuli, both ultimately activate a group of enzymes known as caspases, which are cysteine aspartate-specific proteases. These caspases act as the key executors of apoptosis by cleaving various cellular proteins, ultimately leading to programmed cell death [38,39]. PPARγ promotes apoptosis in lung cancer cells by influencing the key steps in the intrinsic and extrinsic apoptosis pathways, which increase the levels of pro-apoptotic proteins like Bax and Bad, while decreasing the levels of anti-apoptotic proteins such as Bcl-2 and Bcl-XL. These changes boost the activity of caspases-3 and -9, resulting in the release of cytochrome c (Cyt c) from mitochondria [11]. In NSCLC cells, PPARγ activation by KR-62980, a novel PPARγ agonist, or rosiglitazone, enhances reactive oxygen species (ROS) generation through proline oxidase (POX), resulting in the induction of apoptosis [40]. In EGFR-TKI-resistant lung adenocarcinoma cells, the PPARγ agonist, efatutazone, has been shown to trigger both apoptosis and cell cycle arrest via modulating the PPARγ/phosphatase and tensin homolog (PTEN)/AKT axis [41]. Moreover, troglitazone-induced PPARγ activation was shown to phosphorylate extracellular-signal-regulated kinase (ERK1/2), leading to mitochondrial-dependent apoptosis in NCI-H23 lung cancer cells. It also upregulates Growth Arrest and DNA Damage (*GADD153*), a gene associated with DNA damage response, which results in endoplasmic reticulum stress and growth arrest in NSCLC [42,43].

Additionally, PPARγ activation enhances tumor-necrosis-factor-related apoptosis-inducing ligand (TRAIL)-mediated apoptosis in lung adenocarcinoma cells by promoting autophagy flux [44]. The eukaryotic cell cycle consists of the G0, G1, S, G2, and M phases, with DNA replication taking place in the S phase and cell division occurring in the M phase [45]. The regulation of the cell cycle relies on a set of conserved mechanisms, including cyclins, cyclin-dependent kinases (CDKs), G1-S phase transcriptional control, and several checkpoints that monitor DNA damage, replication stress, and spindle assembly [46]. Studies have shown that PPARγ plays a critical role in halting lung cancer cell growth by interfering with the cell cycle. For example, troglitazone activates PPARγ and causes cell cycle arrest at the G0/G1 phase by decreasing cyclins D and E [45]. In addition, PPARγ agonists, prostaglandin J2 (PGJ2), ciglitazone, troglitazone, and GW1929 have been shown to inhibit the growth of lung carcinoma cells by upregulating p21 levels and reducing cyclin D1 expression [47].

Epithelial–mesenchymal transition (EMT) is a key event where epithelial cells lose their polarity and cell junctions, gaining invasive and migratory properties [48]. During tumor progression, the function and expression of the epithelial cell–cell adhesion molecule E-cadherin are often lost, while the expression of the mesenchymal adhesion molecule N-cadherin is upregulated. This loss of E-cadherin, whether at the gene or protein level, is commonly observed in the advancement of many epithelial cancers [49,50]. EMT is one of the hallmarks of metastasis, which is responsible for 90% of cancer-related deaths and involves cancer cells acquiring traits like uncontrolled proliferation, immune evasion, resistance to cell death, angiogenesis, invasiveness, and survival in the bloodstream [51]. To that end, PPARγ inhibits lung cancer metastasis by regulating EMT-related molecules. Activation of PPARγ enhances the expression of the epithelial marker, E-cadherin, and reduces mesenchymal markers (N-cadherin, Snail, and fibronectin), while downregulating matrix metalloproteinase 9 (MMP-9) and heparanase (HPA) [11,52,53]. In addition, PPARγ also suppresses invasion-related proteins, like intercellular adhesion molecule 1 (ICAM-1), and chemokine receptor type 4 (CXCR4), involved in EMT [11,54].

Ferroptosis represents another form of regulated cell death (RCD) initiated by iron-dependent lipid peroxidation via the production of reactive oxygen species (ROS) [55]. Cells undergoing ferroptosis exhibit necrosis-like features, including plasma membrane rupture and shrunken mitochondria with diminished cristae [56]. This process is primarily driven by the accumulation of lipid ROS but occurs independently of caspase activity [57]. While induction of ferroptosis is one of the mechanisms of many anticancer agents, pioglitazone has been shown to inhibit ferroptosis to protect against ionizing-radiation-induced testicular damage, resulting in improved spermatogenesis [58]. However, whether pioglitazone targets ferroptosis to mediate anticancer effects against lung cancer is not yet known.

Moreover, angiogenesis, the formation of new blood vessels from existing ones, is a vital process in both health and disease states [59]. In lung cancer, angiogenesis supports the survival and expansion of tumor cells by delivering essential nutrients and oxygen. This process not only promotes tumor growth but also aids in its spread to surrounding tissues and distant sites and is governed by a delicate balance between the pro-angiogenic and antiangiogenic factors [60,61,62].

While PPARγ activation in inhibiting lung cancer growth is widely accepted, its role in regulating tumor immunity is controversial, likely due to the complex nature of the tumor microenvironment (TME), which includes cancer cells, stromal cells, endothelial cells, and immune cells like macrophages and myeloid-derived suppressor cells (MDSCs) [63]. Accumulating evidence has shown that PPARγ exerts pro-cancer effects in immune cells. For example, PPARγ in myeloid cells has been shown to promote cancer progression and metastasis [64,65]. Studies by Li and colleagues demonstrated that PPARγ activation has context-dependent effects in lung cancer, showing antitumor actions in cancer cells but pro-tumor effects in the tumor microenvironment [64]. Using two immunocompetent mouse models—an orthotopic lung implantation model (CMT/167-luc murine adenocarcinoma cells injected into the left lung) and a subcutaneous flank model (CMT/167-luc cells injected subcutaneously), the authors demonstrated that systemic administration of pioglitazone (0.05% in chow) unexpectedly increased the incidence and number of secondary metastases to the lung, liver, and brain without affecting primary tumor size. Mechanistically, while PPARγ activation in tumor cells reduced invasiveness and promoted differentiation, in myeloid-derived macrophages, it induced the polarization of M2 macrophages as marked by an elevated arginase I expression, which supports angiogenesis, matrix remodeling, immune suppression, and metastatic spread. Bone marrow transplantation experiments using LysM-Cre PPARγ^flox/flox mice confirmed that myeloid-specific PPARγ is required for pioglitazone’s pro-metastatic effects. The authors emphasized that their findings contrast with earlier studies reporting consistent antitumor effects of thiazolidinediones. They noted that retrospective clinical data indicating a reduced incidence of lung cancer among diabetic patients had excluded individuals with pre-existing cancer. Moreover, the authors compared their results to similar context-dependent effects observed in colon cancer models and concluded that the clinical efficacy of PPARγ agonists depends on the balance between their antitumor activity in epithelial cancer cells and their pro-tumor activity in immune cells within the tumor microenvironment [64,65]. This underscores the importance of cell-type specificity and tumor stage in therapeutic targeting of PPARγ pathways.

## 3. Implications of Pioglitazone and Pioglitazone-Based Combination Approaches for NSCLC

### 3.1. Evidence from In Vitro and In Vivo Studies

Several studies have evaluated the effects of pioglitazone in NSCLC using various experimental models ranging from in vitro to in vivo studies. Below, we logically discuss the experimental evidence exploring pioglitazone’s effects on metabolic pathways, chemical-carcinogen-induced tumorigenesis, the targeting of cell signaling pathways, and then its combination with other therapeutic agents.

#### 3.1.1. Pioglitazone’s Effects on Metabolic Pathways

A study was conducted to assess how sumoylation of PPARγ regulates lipid metabolism and enhances tumor suppression in lung cancer cells. The authors utilized H1770, H3255, A549, H2347, Calu6, a genetic pair-matched set of H1993 and H2073, HEK 293 and HBEC cell lines, and a lung xenograft mouse model. The study found that pioglitazone-induced PPARγ not only promoted lipid biosynthesis in a normal serum medium but also induced de novo lipid biosynthesis when the cells were analyzed in a lipid-depleted serum medium. A specific cell line, A549, showed both lipid biosynthesis and β oxidation. Notably, sumoylated PPARγ, which refers to the post-translational modification of the PPARγ by the Small Ubiquitin-like Modifier (SUMO) protein, was shown to play a crucial role in lipid metabolism that was seen in PPARγ WT, but not in a PPARγ-mutated model [35]. Similar results were seen in in vivo studies, when A549 lung cancer cells were injected into athymic nude mice and treated with pioglitazone. The study also focused on the role of redox balance and demonstrated that fatty acid synthesis depletes NADPH levels and increases ROS, which ultimately suppresses tumor progression capacity of lung cancer cells. The study provides key insights into the role of sumoylated PPARγ in regulating lipid metabolism and tumor suppression by promoting lipid biosynthesis and β-oxidation in lung cancer cells. Furthermore, it imparts the relationship between fatty acid synthesis and redox imbalance, where decreased NADPH and increased ROS can contribute to tumor suppression [35]. A summary of studies highlighting pioglitazone’s role and mechanisms with or without other therapeutic approaches is given in Table 1.

Enzymes and transcription factors frequently establish unexpected interactions in the complex network of cancer metabolism that either promote tumor growth or inhibit it. In this context, Hua and colleagues conducted a study to examine the regulatory connection between ALDH1A3 and PPARγ in the metabolism of lung cancer. The microarray dataset of 78 lung cancer cell lines revealed a substantial positive connection between PPARγ and ALDH1A3, a major enzyme involved in retinoic acid synthesis and NADH generation. To test this, they used HBEC cells (engineered for tetracycline-induced PPARγ expression), and H1993 (PPARγ-positive) and H1299 (PPARγ-negative) lung cancer cell lines. The ALDH1A3 promoter included PPAR response elements (PPREs), according to an in silico promoter analysis conducted with NUBIScan software (https://academic.oup.com/mend/article-abstract/16/6/1269/2741719?redirectedFrom=fulltext, accessed on 18 September 2025). ALDH1A3 was found to be a direct inverse target of PPARγ, as demonstrated by ChIP experiments [62]. Both HBEC and H1993 cell lines showed dose-dependent suppression of ALDH1A3 expression after being treated with pioglitazone. In particular, in H1993 cells, the formation of 4HNE-protein adducts suggested that this inhibition resulted in substantial lipid peroxidation. Lipid peroxidation resulting from pioglitazone-mediated inhibition of ALDH1A3 exacerbates oxidative stress. The study showed that inhibiting ALDH suppresses the growth of lung cancer cells. Pioglitazone only inhibited growth in PPARγ-positive H1993 cells in colony formation experiments, but 4-diethylaminobenzaldehyde (DEAB), an ALDH inhibitor, decreased proliferation and sensitized the cells to pioglitazone. Increased ROS, PARP breakage, and reduced cyclin A and B1 indicate cell cycle arrest and apoptosis, which were associated with the cytotoxic effects of pioglitazone. The study concludes by showing that PPARγ directly binds and inhibits ALDH1A3, preventing the generation of retinoic acid and inducing oxidative stress via lipid peroxidation. Overall, ALDH1A3 has been identified as a novel metabolic target of PPARγ that has therapeutic implications, and this regulatory pathway is crucial in restricting the growth of lung cancer cells [66].

#### 3.1.2. Pioglitazone Effects on Chemical Carcinogens-Induced Tumorigenesis

A study examined whether pioglitazone could help prevent lung cancer by halting the progression of squamous dysplasia induced by N-nitroso-trischloroethylurea (NTCU, a chemical known to trigger precancerous lung changes). Researchers used female FVB/N mice (4–5 weeks old; *n* = 36/group at the start) and exposed them to NTCU because of their known susceptibility to NTCU. Animals were randomized into three groups: acetone as a vehicle control, NTCU alone, and NTCU + pioglitazone (0.05% in diet), with pioglitazone administration beginning two weeks prior to NTCU exposure. NTCU (20 mM in acetone) was topically applied twice weekly to shaved dorsal skin for 32 weeks. The authors noted that NTCU exposure was associated with toxicity, weight loss, and animal attrition, the recognized limitations of this model. Health assessments led to temporary treatment suspensions and euthanasia of four mice in the NTCU group and five mice in the NTCU + pioglitazone group due to severe toxicity. Tissue samples were analyzed for histological changes using H&E staining and immunohistochemistry (IHC), while immunofluorescence was employed to track lesion development, basal cell expansion [Keratin 5 (KRT5), KRT14, and p63], and the loss of normal airway cell markers [Clara cell secretory protein (CCSP+), and acetylated tubulin (ACT+)]. Gene expression changes related to EMT and dysplasia persistence such as Desmoglein-3 (*DSG3*), and Polo-like kinase 1(*PLK1*)] were quantified by qPCR. The data demonstrated that NTCU exposure significantly increased the incidence of high-grade dysplasia and induced EMT-associated molecular changes such as reduced epithelial marker E-cadherin (*p* = 0.007) and increased mesenchymal markers vimentin (*p* = 0.003) and FN1, along with elevated basal cell markers KRT14 (*p* = 0.0001), KRT5 (*p* = 0.06), and p63 (*p* = 0.0004). Pioglitazone treatment significantly reduced high-grade dysplasia (*p* < 0.05 vs. NTCU), decreased KRT14 (*p* = 0.0001) and p63 (*p* = 0.001) to near-control levels, restored E-cadherin (*p* = 0.02) and lowered vimentin (*p* = 0.01). It also reduced DSG3 mRNA (*p* = 0.015), modestly lowered *PLK1* mRNA (*p* = 0.17), and reversed the NTCU-induced loss of club (*p* = 0.01 trachea, *p* = 0.02 bronchus) and ciliated cells (*p* = 0.001 trachea, *p* = 0.03 bronchus). Overall, this study demonstrated that pioglitazone exerts a chemopreventive effect against NTCU-induced high-grade lesions, likely through reversal of EMT and restoration of epithelial differentiation [67].

In an effort to explore novel strategies for lung cancer prevention, Wang and colleagues investigated the effects of pioglitazone on lung tumor development using both in vitro and in vivo models [68]. The aim was to assess pioglitazone’s ability to inhibit the progression of established lung tumors, specifically adenocarcinoma and squamous cell carcinoma (SCC). To that end, human NSCLC cell lines A549 and H1299 were employed to examine growth inhibition and apoptotic gene expression, while in vivo studies were carried out using mouse models of lung adenocarcinoma (induced by vinyl carbamate) in both p53 wild-type (p53wt/wt) and p53 mutant (p53wt/Ala135Val) backgrounds, as well as an SCC model induced by NTCU in female NIH Swiss mouse. A total of 138 mice were divided into multiple experimental and control groups. In the adenocarcinoma arm, 78 female A/J mice aged 6 weeks (both p53wt/wt and p53wt/Ala135Val genotypes) were randomly divided into four groups: p53wt/wt vehicle control, p53wt/wt + pioglitazone, p53wt/Ala135Val vehicle control, and p53wt/Ala135Val + pioglitazone. All received two intraperitoneal doses of vinyl carbamate (0.32–0.35 mg) one week apart, and treatment with pioglitazone (15 mg/kg by oral gavage) began 8 weeks later, when microscopic adenomas had formed, continuing for 12 weeks. In the SCC arm, 60 female NIH Swiss mice were used, randomly assigned to NTCU vehicle control and NTCU + pioglitazone groups. SCC was induced with topical N-nitroso-tris-chloroethylurea (0.03 mol/L in acetone, 100 μL) applied to shaved dorsal skin twice weekly for 32 weeks; pioglitazone began at week 8 and continued for 24 weeks. The NTCU protocol, although effective, had major disadvantages including long duration, high toxicity, skin irritation, weight loss, and animal attrition, making it labor-intensive. Endpoints included tumor incidence, multiplicity, tumor load, histopathology, active caspase-3 apoptosis index, Ki-67 proliferation index, and in vitro testing on NSCLC cell lines (A549, H1299). Pioglitazone significantly reduced lung adenocarcinoma tumor load by 63.9% in p53wt/wt mice (*p* = 0.0005) and 49.6% in p53wt/Ala135Val mice (*p* = 0.0069), without affecting multiplicity, and converted invasive adenocarcinomas to non-invasive adenomas. In SCC, pioglitazone decreased carcinoma burden by 35% (*p* < 0.05) and increased normal bronchial epithelium by 24%. Apoptosis was markedly induced in both models, with active caspase-3–positive cells in SCC lesions increasing from 0.41 ± 0.11 to 0.87 ± 0.12 per field (*p* = 0.008), and proliferation was reduced in p53wt/wt adenocarcinomas, with Ki-67 staining decreasing by 31% (*p* = 0.0003) [68]. In immunohistochemical analysis, Ki-67 staining revealed reduced tumor cell proliferation in p53wt/wt mice, but not in p53 mutant mice, indicating a p53-dependent effect, while caspase-3 staining showed increased apoptosis in all models, highlighting apoptosis as a primary mechanism. These findings demonstrated that pioglitazone’s mechanism of action is primarily through the induction of apoptosis and, to a lesser extent, inhibition of proliferation, offering promising potential for its use as a chemopreventive agent across multiple NSCLC subtypes with p53 statuses [68].

In a study, the role of PPARγ was investigated using an A/J mouse model that was treated with the carcinogen 4-(methylnitrosamino)-1-(3-pyridyl)-1-butanone (NNK; 100 mg/kg, intraperitoneally) to induce pulmonary tumors [69]. The mice were randomly divided into four groups, saline as a vehicle control, NNK-alone, PGZ-alone, and NNK + pioglitazone (PGZ; 20 mg/kg/day, gavage, starting at week 50), with 10 mice in each of groups 1 and 2 being sacrificed every four weeks from weeks 26 to 54 and 10 mice in each of groups 3 and 4 being sacrificed at 54 weeks of treatment. The experiment included multiple assays to evaluate PPARγ expression and activity. Real-time PCR analysis revealed a significant upregulation of *PPARγ* mRNA in NNK-treated lung tissue compared to control samples (*p* < 0.05). Immunohistochemical staining demonstrated strong nuclear PPARγ expression in NNK-induced tumors relative to normal lung tissue across all time points (weeks 26–54; *p* < 0.001), which was further confirmed by Western blot analysis showing increased nuclear PPARγ protein levels. Despite this elevated expression, PPARγ transcriptional activity, measured by PPARγ Transcription Factor Assay kits, was significantly reduced in NNK-treated lung tumors compared to controls (*p* < 0.05 at all time points). ELISA assays showed that endogenous PPARγ ligands, 15(S)-HETE and 13(S)-HODE, were markedly decreased in NNK-treated tumor tissues (*p* = 0.0004 and *p* = 0.0035, respectively) and in human lung cancer samples (*p* = 0.011 and *p* = 0.022, respectively). Treatment of a subset of NNK-exposed mice with the synthetic PPARγ agonist pioglitazone significantly inhibited lung tumor formation, restored ligand levels [15(S)-HETE (*p* = 0.0079); 13(S)-HODE (*p* = 0.0082)], and increased PPARγ transcriptional activity (*p* = 0.0011). Overall, these results highlighted the complex role of PPARγ in lung carcinogenesis, characterized by increased expression but reduced functional activity during tumor progression, and demonstrated the therapeutic potential of pioglitazone in restoring PPARγ signaling and suppressing tumor development [69].

In the above studies, variability in mouse strains, carcinogen dosing regimens, and study endpoints can significantly influence outcomes in preclinical lung tumorigenesis models. For instance, A/J mice are highly susceptible to chemically induced lung tumors, developing predominantly adenomas and adenocarcinomas following exposure to NNK, vinyl carbamate, or benzo[a]pyrene (BaP) [70]. In contrast, C57BL/6 and BALB/c mice exhibit markedly lower tumor incidence under similar conditions, illustrating clear strain-dependent susceptibility [71,72]. Similarly, NTCU, although primarily used to induce squamous lesions, can also be used for NSCLC studies in susceptible strains such as FVB/N and NIH Swiss, as it can induce lesions that progress along the adenocarcinoma spectrum [73]. Moreover, immune-deficient strains such as nude or SCID mice modulate tumor progression and multiplicity due to impaired immune surveillance [74]. The dosing regimen, including route, frequency, and cumulative exposure, further affects tumor latency, multiplicity, and grade, with higher doses sometimes increasing toxicity [73]. Finally, variation in study endpoints, such as tumor incidence, multiplicity, histologic grade, and molecular marker expression, complicates cross-study comparisons. Together, these factors underscore the importance of carefully selecting mouse strain, carcinogen, and study design to accurately model NSCLC and interpret preclinical results effectively.

#### 3.1.3. Pioglitazone’s Effects on the Targeting of Cell Signaling Pathways

A study investigated the role of PPARγ activation in lung cancer treatment through both in vitro and in vivo experiments, demonstrating its potential as a therapeutic target. In vitro studies on A549 NSCLC cells showed that PPARγ ligands (troglitazone and pioglitazone) significantly suppressed the secretion of pro-angiogenic ELR + CXC chemokines [i.e., IL-8/CXCL8, epithelial neutrophil-activating protein 78 (ENA-78/CXCL5), and growth-regulated oncogene alpha (Gro-α/CXCL1)], as confirmed by ELISA assays. This suppression was further validated using a transient transfection assay with an active PPARγ construct (VP16-PPAR-γ), demonstrating direct regulation of chemokine expression [68]. Additionally, PPARγ activation inhibited nuclear factor kappa B (NF-κB) transcriptional activity, as shown through TransAM reporter assays. These findings were further confirmed with an NF-κB inhibitor, pyrrolidinecarbodithioic acid (PDTC), which also reduced the levels of these three chemokines, CXCL8, CXCL5, and CXCL1. In addition, conditioned media from treated A549 cells exhibited reduced chemotactic activity in human microvascular endothelial cells (HMVECs), indicating impaired tumor-induced angiogenesis. In in vivo studies, SCID mice were xenografted with A549 cells and treated daily with PPARγ ligands (troglitazone: 200 mg/kg and pioglitazone: 25 mg/kg) for 8 weeks. The data showed a ~65% reduction in tumor growth. Moreover, the immunohistochemical staining for factor VIII, which is an endothelial marker, revealed a 60% decrease in blood vessel density, confirming an antiangiogenic effect. The in vivo results correlated with in vitro findings, reinforcing that PPARγ activation suppresses both tumor proliferation and angiogenesis by inhibiting chemokine production and NF-κB activity. Overall, this study provides compelling evidence that PPARγ ligands can significantly hinder NSCLC progression by targeting both tumor growth and its ability to promote blood vessel formation, underscoring its potential for further therapeutic development. However, the study primarily focused on the cell line A549, which may limit the relevance to other types of NSCLC [75].

The study examined the effects of thiazolidinediones (TZDs), specifically pioglitazone and rosiglitazone, on prostaglandin E2 (PGE2) levels in A427 and A549 NSCLC cell lines. The aim was to determine whether these TZDs could suppress PGE2 production through a cyclooxygenase-2 (COX-2) independent mechanism. NSCLC cells were treated with TZDs, along with arachidonic acid, to stimulate PGE2 production. PGE2 levels were measured using immunoassays, and COX-2 expression was assessed via Western blot and ELISA assay. The findings revealed that TZD treatment did not affect COX-2 levels, indicating that PGE2 reduction occurred through a COX-2 independent pathway [69]. Additionally, no changes were observed in the expression of the three enzymes involved in PGE2 synthesis: PGES1, cytosolic PGES, and microsomal PGES2. Significantly, pioglitazone and rosiglitazone increased the expression of 15-hydroxyprostaglandin dehydrogenase (15-PGDH), which inactivates PGE2. The importance of 15-PGDH was confirmed through small interfering RNA (siRNA) targeting, which inhibited the TZD-mediated reduction of PGE2. Experiments using dominant-negative PPARγ overexpression and the PPARγ antagonist GW9662 indicated that the suppression of PGE2 was independent of PPARγ activation. Overall, the study suggests that TZDs can reduce tumor-derived PGE2, offering a potential therapeutic strategy for lung cancer management without the cardiovascular risks associated with direct COX-2 inhibition. Limitations include the reliance on only two cell lines, which may not fully represent lung cancer variability, and the need for further in vivo validation of these findings. This research provides valuable insights into how TZDs might modulate PGE2 metabolism in lung cancer [76].

Metastasis is a complex process involving genetic and epigenetic alterations that activate or silence tumor-related genes [77]. A study by Yoo and colleagues aimed to explore the underlying mechanisms behind brain metastasis in NSCLC, focusing on the role of EMT and evaluating pioglitazone for its potential to modulate EMT in an experimental murine model. To that end, NCI-H358 human lung adenocarcinoma cells were injected into the right frontal lobe of nude mice, and mice received pioglitazone either before, after, or both before and after inoculation of tumor cells. It was found that E-cadherin expression increased with pioglitazone exposure, with higher expression seen in the group where mice were treated with pioglitazone both pre- and post-inoculation of tumor cells. In addition, mesenchymal markers, MMP9 and fibronectin, were decreased in pioglitazone-treated groups, particularly with pretreatment, and fibronectin expression was reduced proportionally to pioglitazone exposure. In conclusion, the study demonstrated that E-cadherin loss is significantly associated with brain metastasis in NSCLC and may serve as a predictive marker. EMT plays a critical role in tumor invasiveness and metastasis, and pioglitazone enhanced E-cadherin expression while suppressing mesenchymal markers, thereby inhibiting EMT and reducing the metastatic potential of NSCLC [78].

A study on pioglitazone’s effects on NSCLC employed a comprehensive array of investigations to evaluate its antitumor activity. The authors utilized multiple NSCLC cell lines (A549, H1299, H460, H1975, HCC827), normal bronchial epithelial cells (Beas2B), and ex vivo 3D adenocarcinoma cultures. Methods included 3-(4,5- dimethylthiazol-2-yl)-2,5 diphenyltetrazolium bromide (MTT) for viability assay, annexin V/7-Amino-Actinomicin D (V/7-AAD) flow cytometry for apoptosis, Western blotting, qRT-PCR, microarray profiling, and sea horse analysis to assess glycolysis and oxidative metabolism. Pioglitazone reduced cancer cell viability and induced apoptosis via caspase-3 activation while selectively sparing normal cells. Gene expression profiling showed the downregulation of MAPK, Myc, Ras, and EMT regulators TGFβR1 and Suppressor of Mothers Against Decapentaplegic (SMAD3) and the upregulation of pro-apoptotic genes (CASPASE 4/5 (CASP4/5), B-cell translocation gene 1 (BTG1), and programmed cell death 4 (PDCD4). Pioglitazone also reduced the phosphorylation of EGFR, AKT, and MAPK and suppressed glycolysis by lowering extracellular acidification rate (ECAR), GLUT1, glucose-6-phosphate dehydrogenase (G6PD), transketolase (TKTL1), and Solute carrier family 15 (SLC15A) expression. Altogether, these findings indicate that pioglitazone regulates multiple cellular activities, including suppressing cell proliferation, inducing apoptosis, impeding invasiveness, and reprogramming metabolism, supporting its potential as a therapeutic agent for treating NSCLC [17].

#### 3.1.4. Pioglitazone’s Effects in Combination Therapy Efficacy

A study was conducted by Seabloom and colleagues with the aim of evaluating the efficacy of pioglitazone and metformin, both alone and in combination, on lung adenoma formation in a B{a}P-induced carcinogenesis model in female A/J mice and Beas-2B bronchial epithelial cells for proliferation assays. In the dose-finding study, 192 mice were randomized into 6 groups of 32 animals each, receiving a control diet, pioglitazone (15 mg/kg/day), metformin (500 or 850 mg/kg/day), or combinations of pioglitazone with metformin. Statistical analyses were performed using one-way ANOVA with Dunnett’s post hoc test to identify statistically significant differences between control and treatment groups or between combination and single-agent groups. For immunohistochemistry (IHC) data, staining intensity scores were compared across groups using the Kruskal–Wallis nonparametric test to evaluate differences in the expression of cyclin D1 and Ki-67 between the treated and control groups. The authors observed that after 15 weeks, metformin alone significantly reduced adenoma counts in a dose-dependent manner (500 mg/kg/day: 35.7% reduction, *p* = 0.001; 850 mg/kg/day: 58.3% reduction, *p* ≤ 0.0001), pioglitazone alone reduced adenomas by 29.3% (*p* = 0.0098), and the combinations produced 43.5–53.5% reductions (*p* = 0.004 to <0.0001). In the intervention-stage chemoprevention study, 224 mice were randomized assigned (28 mice/group) to test the effects of early-stage intervention of metformin and/or pioglitazone started 7 days after the last dose of B[a]P and late-stage intervention started 8 weeks after the last dose of B[a]P administration. Early intervention showed robust reductions (pioglitazone 15 m/kg/day: 32%, *p* = 0.0023; metformin 850–1000 mg/kg/day: 65–71%, *p* < 0.0001; combination: 70%, *p* < 0.0001), whereas late intervention was less effective (pioglitazone: 17%, non-significant; metformin 850 mg/kg/day: 38%, *p* = 0.0007; and combination: 44%, *p* < 0.0001). IHC analyses for cyclin D1 and Ki-67 showed no significant differences between the treated and control groups. In in vitro studies, pioglitazone (10 µM) reduced Beas-2B proliferation, while metformin (5–20 mM) had a minimal additive effect [79]. In conclusion, metformin does induce a significant reduction in adenoma counts alone but less significantly with the combination of pioglitazone. This research underscores the importance of exploring further mechanisms, particularly in the context of cancer prevention and the interplay with other agents like pioglitazone.

Along similar lines, Fu and colleagues evaluated the chemopreventive effect of pioglitazone alone or its combination with aerosolized budesonide against B(a)P-induced lung tumorigenesis in female A/J mice [80]. Seventy-two mice received a single intraperitoneal dose of B[a]P (100 mg/kg) and, two weeks later, were divided into six groups (twelve mice/group): solvent control, gavage control, solvent + gavage control, budesonide alone, pioglitazone alone, and their combination. Budesonide was delivered by aerosol (initially at a dose of ~209 µg/kg, which was later reduced to ~157 µg/kg body weight daily for 20 weeks), while pioglitazone was given by oral gavage (10 mg/kg, 5 days/week for 20 weeks). The study used two main statistical approaches. In animal experiments, one-way ANOVA with Tukey’s post hoc test was used to compare the tumor multiplicity and tumor load across control, budesonide, pioglitazone, and combination groups. In cell culture, a linear regression model assessed drug interactions, including individual effects of budesonide (B_i_), pioglitazone (P_j_), and their interaction (BP_ij_), along with control (C_k_). Both drugs had significant individual effects (*p* < 0.05), while their interaction was not significant (*p* = 0.45), indicating independent, additive inhibitory effects on cell growth. Budesonide alone reduced tumor multiplicity by 57% (*p* < 0.05) and tumor load by 78% (*p* < 0.05), and pioglitazone alone decreased tumor load by 63% (*p* < 0.05) but did not affect tumor multiplicity, while their combination led to a ~90% reduction in tumor load (*p* < 0.05 vs. control). Tissue assays confirmed that aerosolized budesonide concentrated specifically in the lung without systemic distribution, whereas pioglitazone was detected in multiple tissues. The in vitro studies using A549 and H1299 human NSCLC cell lines demonstrated that both budesonide and pioglitazone independently inhibited cell proliferation in a dose- and time-dependent manner, with additive but not synergistic effects seen wit the combination of budesonide and pioglitazone. The authors emphasized that chemoprevention using combinations of drugs acting on distinct pathways could enhance efficacy while minimizing toxicity. Aerosol delivery was highlighted as an advantageous method because it maximizes lung targeting while reducing systemic exposure. In conclusion, pioglitazone reduced tumor size but not tumor number, yet its combination with budesonide was highly effective, suggesting additive inhibition of tumor progression. Importantly, no antagonistic interactions were observed. Limitations include the use of a single carcinogen-induced mouse model, and the authors noted the need for validation in diverse models and eventually human trials [80].

Studies by Kiran and colleagues conducted an in vivo study in male Balb/C mice to evaluate the anticancer potential of combining pioglitazone and celecoxib (COX-2 inhibitor) against nicotine-derived nitrosamine ketone (NNK)-induced NSCLC. Sixty mice were divided into six groups of ten each: sham control (saline, no NNK), disease control (NNK 10 mg/kg intratracheal), pioglitazone alone at 10 mg/kg and 20 mg/kg orally, and two combination groups receiving pioglitazone with celecoxib at a low dose (10 mg/kg + 25 mg/kg, p.o.) or a high dose (20 mg/kg + 50 mg/kg, p.o.) for 12 weeks after tumor induction. Parameters assessed included body weight, mean survival time (MST), percentage increase in lifespan (%ILS), tumor weight, and histopathology. Statistical analyses were evaluated by one-way ANOVA followed by Dunnett’s multiple comparison test. The results showed that disease control mice exhibited significant weight loss and a reduced MST of 19.2 weeks, while pioglitazone alone increased MST to 29.3 and 30.1 weeks and %ILS to 52.6% and 56.8%, respectively. The combination treatment further improved outcomes, with MST values of 30.7 and 31.4 weeks, and %ILS of 59.9% and 63.5% (*p* < 0.05 vs. disease control). Notably, 30% of mice in the high-dose combination group survived to week 32, whereas no disease control mice survived beyond week 24. Lung weights were significantly higher in disease controls (*p* < 0.05 vs. sham) but were reduced in all treatment groups, though not always significantly. Histopathological analysis revealed that disease control lungs exhibited disrupted alveolar architecture and adenocarcinoma features, while treated groups showed dose-dependent preservation of lung architecture, most pronounced in the high-dose combination group. The authors noted the absence of a celecoxib-only group as a limitation and emphasized the need for mechanistic studies and clinical validation. Overall, the findings suggest that combined pioglitazone and celecoxib therapy exerts greater tumor-inhibitory and survival benefits than either agent alone, supporting their potential as a chemopreventive strategy against NNK-induced NSCLC [21].

In the above preclinical studies, each treatment group included approximately 10–32 animals, and various statistical methods and measures of variability were employed to evaluate differences among groups. Despite some limitations, the reported analyses demonstrate biologically meaningful and statistically robust effects of combination therapy compared with single-agent treatment.

Overcoming drug resistance remains one of the biggest challenges in treating EGFR-mutant NSCLC patients. While the T790M EGFR mutation drives acquired TKI resistance, PTEN loss has also emerged as a key factor in both primary and acquired drug resistance [81,82]. To and colleagues investigated whether PPARγ agonists could resensitize PTEN-deficient NSCLC cells to the EGFR tyrosine kinase inhibitor gefitinib. Using in vitro models, the authors tested HCC827 (EGFR-mutant, PTEN-intact, TKI-sensitive), H1650 (EGFR-mutant, PTEN-deficient, TKI-resistant), a normal bronchial epithelial line (BEAS-2B), and HCC827 cells with stable PTEN knockdown via shRNA. The drugs studied included rosiglitazone, pioglitazone, and telmisartan, each alone and in combination with gefitinib. Cell proliferation, apoptosis, and autophagy were quantified, and drug interactions were analyzed using CompuSyn software (https://www.combosyn.com/index.html, accessed on 18 September 2025) to generate Combination Index (CI)–Fraction affected (Fa) plots. Interactions were classified as synergistic (CI < 1), additive (CI = 1), or antagonistic (CI > 1). Statistical analysis included Student’s *t*-test and Bonferroni correction for multiple comparisons, ensuring robust quantitative validation and biological reproducibility of the observed effects. In PTEN-deficient H1650 cells, gefitinib (6 µM) alone induced only 8.2 ± 2.2% apoptosis, while rosiglitazone (25 µM), pioglitazone (25 µM), and telmisartan (4 µM) alone caused 17.2 ± 2.6%, 15.3 ± 2.9%, and negligible apoptosis, respectively. However, combining gefitinib with rosiglitazone or pioglitazone resulted in a dramatic increase in apoptosis to 48.2 ± 4.2% or 46.5 ± 4.5%, respectively (*p* < 0.017 vs. gefitinib alone), while gefitinib plus telmisartan yielded a more modest but still significant effect (28.6 ± 3.2% apoptosis, *p* < 0.05). Combination index analysis confirmed strong synergy for gefitinib with rosiglitazone and pioglitazone (CI ~ 0.3–0.4 at 50% growth inhibition) and concentration-dependent sensitization by telmisartan. Mechanistically, PPARγ agonists restored PTEN protein expression, reduced phosphorylated Akt, and markedly induced autophagy (e.g., GFP-LC3 puncta positive cells rose to ~84%, *p* < 0.01). Importantly, genetic silencing of ATG5 abolished synergy: the gefitinib–rosiglitazone combination apoptosis fell from 46.4 ± 3.9% in control cells to 28.4 ± 3.1% in ATG5-silenced cells, and CI shifted from <1 (synergistic) to ~1 (additive). No synergy was observed in PTEN-intact HCC827 cells unless PTEN was knocked down, underscoring PTEN loss as the determinant of response. In conclusion, PPARγ agonists resensitize PTEN-deficient, EGFR-mutant NSCLC to gefitinib through a PPARγ-dependent induction of autophagy, supporting a biomarker-driven therapeutic strategy to overcome TKI resistance [83].

**Table 1:** Summary of the in vitro and in vivo studies defining the role and mechanisms of pioglitazone as monotherapy and pioglitazone-based combination approaches in TNBC.

Drug	Cell Lines/ Mouse Models	Targets	Findings	Refs.
Pioglitazone	H1770, H3255, A549, H2347, Calu6, H1993, H2073, HEK 293 and HBEC Athymic mice	PPARγ	Pioglitazone-mediated sumolyation of PPARγ induced tumor suppression via disrupting redox balance	[35]
Pioglitazone	HBEC, H1993, H1299	PPARγ	Pioglitazone inhibited ALDH1A3 and production of aldehydes and induced ROS generation, which resulted in the restriction of cell growth	[66]
Pioglitazone	FVB/N	PPARγ	Pioglitazone caused a reduction in high-grade dysplasia, reversal of EMT-associated gene changes, and complete elimination of basal cells in bronchial epithelium	[67]
Pioglitazone	A549, H1299, p53 wild-type (p53wt/wt) and mutant (p53wt/Ala135Val) mice. Female NIH Swiss mice.	Caspase-3, Ki-67	Pioglitazone suppressed lung cancer progression and caused induction of apoptosis	[68]
Pioglitazone	A/J mice	PPARγ	Pioglitazone inhibited the formation of lung tumors	[69]
Pioglitazone	A549 Cells SCID mice	PPARγ	Pioglitazone resulted in the suppression of tumor proliferation and pro-angiogenic chemokines (ELR + CXC)	[75]
Pioglitazone and Rosiglitazone	A427 and A549	PGE2	PPARγ agonists reduced PGE2 levels to suppress cancer growth	[76]
Pioglitazone	NCI-H358 cells and Nude mice	E-cadherin	E-cadherin loss is associated with brain metastasis. Pioglitazone enhances E-cadherin expression and suppresses EMT.	[78]
Pioglitazone	H1299, H460, A549, H1975, HCC827, and Beas2B	MAPK, NF-κB, EGFR/AKT, TNFα, TGF β/SMAD, Myc, R-Ras, and Transketolase	Pioglitazone inhibited tumor cell proliferation, reduced invasiveness, induced apoptosis, and modulated cancer cell metabolism	[17]
Pioglitazone and metformin	B(a)P mouse	PPARγ	Combination therapy resulted in significant reduction in adenomas	[79]
Pioglitazone and aerosolized budesonide	A549, H1299 B(a)P mouse,		Combination therapy inhibited lung adenomas and reduced tumor burden	[80]
Pioglitazone and celecoxib	Balb/c mice	NFκB and COX-2	Combination therapy caused reduced lung tissue weight and improved histopathological features, indicating effective tumor suppression	[21]
Pioglitazone and gefitinib	HCC827, H16650, BEAS-2B,	PTEN, Akt, EGFR TKI	Combination therapy induced apoptosis and autophagy to inhibit cell growth	[83]

### 3.2. Evidence from Clinical Studies

Although pioglitazone has shown promising antitumor effects in a wide range of preclinical lung cancer studies, turning the success into meaningful results in humans has been more complicated. Laboratory and animal research, especially in NSCLC models, has consistently demonstrated that pioglitazone, through its activity as a PPARγ agonist, can slow tumor growth, promote cell differentiation, and even prevent progression of early-stage lesions. These encouraging results laid the groundwork for testing the drug in clinical trials, particularly in high-risk individuals where preventative approaches could make a real difference.

In this context, the randomized phase II clinical trial conducted by Keith and colleagues served as an essential attempt to assess pioglitazone’s ability to improve precancerous lung lesions in current and former smokers. The study enrolled 92 participants, comprising 44 current smokers and 48 former smokers. Forty-seven participants were assigned to pioglitazone and forty-five to placebo. Eligible participants had a history of smoking at least 10 packs of cigarettes per day per year and showed evidence of lung damage via sputum cytologic atypia or biopsy-proven endobronchial dysplasia. Subjects received a daily oral dose of 30 mg pioglitazone or placebo for six months. The primary endpoint was a change in the worst (maximum) histological biopsy score (Max), while secondary endpoints included an average biopsy score (Avg), dysplasia index (DI), Ki-67 proliferation index, and inflammation scores. Bronchoscopic biopsies were taken at baseline after treatment. After six months, 76 participants completed follow-up bronchoscopies with 39 participants in the treatment group and 37 participants in the placebo group. Overall, the trial did not show statistically significant improvements in any of the histological endpoints for the pioglitazone-treated group versus placebo. However, a trend towards improved histology and decreased Ki 67 index was observed in former smokers treated with pioglitazone, though these findings did not show statistical significance. Inflammatory scores also suggested mild improvement trends in some subgroups, but no clear correlation with treatment efficacy was established. Adverse events were similar between groups, except lower rates of hyperglycemia were documented in the pioglitazone-treated group. In conclusion, while the study confirmed the safety of pioglitazone in this population, it did not demonstrate any significant histological benefits in lung cancer chemoprevention and warrants further investigation [84]. A summary of clinical studies is highlighted in Table 2.

While preclinical studies in mice and other animal models have shown robust efficacy through modulation of signaling pathways such as MAPK, NF-κB, EMT markers, redox balance, and ALDH1A3, these promising results have not consistently translated into clinical benefits in humans. This discrepancy can be attributed to multiple factors. First, species-specific differences in physiology, drug metabolism, and immune responses can influence drug exposure and target engagement [85]. Second, animal disease models often fail to fully replicate the heterogeneity, complexity, and comorbidities of human disease, leading to overly optimistic preclinical outcomes. Most preclinical oncology studies utilize inbred mouse strains such as C57BL/6 or BALB/c, which are genetically homogeneous. While this reduces variability in experimental outcomes, it often fails to capture the genetic diversity seen in human populations, where differences in genetic background can substantially influence drug metabolism, immune response, and disease progression [86]. Third, dosing regimens in rodents may achieve higher drug concentrations than are feasible or safe in humans, which limits comparable therapeutic effects [87]. Moreover, patient populations enrolled in clinical trials are genetically diverse and exposed to various environmental factors, in contrast to the controlled and uniform conditions in preclinical settings. In the phase II trial by Keith et al., the lack of significant histological improvement may have resulted from suboptimal dosing, absence of biomarker-guided patient selection, and inclusion of a heterogeneous patient cohort, all of which could have obscured potential therapeutic effects. Collectively, these limitations underscore the need for biomarker-driven patient stratification, optimized dosing strategies, and more predictive, genetically diverse disease models to better bridge the translational gap between preclinical promise and clinical efficacy.

**Table 2:** Evidence of pioglitazone’s effects from clinical studies is summarized.

Drug	Study Type/ Population	Assessments	Dosing	Findings	Refs.
Pioglitazone	Randomized phase II trial with the goal of improving precancerous lung lesions in current and former smokers (92 high-risk smokers)	Histology, Ki-67, Inflammation	30 mg	No significant histological improvement, trend toward benefit in former smokers	[87]
Pioglitazone	Pilot window trial in 6 patients with stage IA–IIIA NSCLC (smokers/ex-smokers)	Ki-67 expression; gene expression in airway tissue	45 mg	20% median reduction in Ki-67; all patients showed decreased proliferation; immune gene modulation observed (inflammatory/B cell survival, and increased complement/chemokine signaling	[88]

A pilot study evaluated pioglitazone’s effect as a potential chemopreventive agent in early-stage NSCLC patients. Six patients with stage IA–IIIA NSCLC with a history of heavy smoking received pioglitazone (45 mg/day) for 14–42 days prior to surgical resection. Ki-67 staining was assessed in tumor tissue before and after treatment. Among the five eligible participants, all showed a reduction in Ki-67 expression, with a median decrease of 20% (*p* = 0.06). Additionally, gene expression analysis of normal bronchial epithelium revealed upregulation of complement activation and chemokine signaling pathways and downregulation of inflammatory and B cell survival pathways (GSEA; *p* < 0.05). These findings suggest that pioglitazone may reduce tumor cell proliferation and favorably alter immune-related gene expression, supporting its potential in NSCLC chemoprevention, though further studies with large cohorts are needed [88].

A double-blind, placebo-controlled phase II clinical trial tested the effects of oral pioglitazone for lung cancer chemoprevention in high-risk current and former smokers with sputum cytologic atypia or known endobronchial dysplasia. The trial recruited 92 subjects, of whom 47 subjects were given pioglitazone and 45 were on placebo. After six months of pioglitazone treatment, the endobronchial dysplasia (Max histology score) and Ki-67 index were analyzed as primary/intermediate endpoints. While pioglitazone did not produce a statistically significant improvement in bronchial dysplasia, some lesion-level improvements were seen in former smokers, whereas slight worsening was noticed in current smokers. Overall, the trial did not support the broad chemopreventive use of pioglitazone in that population and concluded that further study is warranted to characterize the significance of pioglitazone in responsive dysplasia [84].

Another single-center pilot clinical trial tested the effect of pioglitazone for the treatment of stage IA to IIB NSCLC patients, with the primary and secondary endpoints being the assessment of tissue biomarkers such as Ki-67, toxicity/safety profile, and tumor metabolic activity by fludeoxyglucose positron-emission tomography (FDG-PET). Patients were planned to receive pioglitazone 45 mg orally once a day, with the duration of treatment (minimum of 2 weeks or a maximum of 6 weeks) determined by standard of care and scheduling of surgery. However, this trial did not report any benefit due to low accrual of patients [89].

While further exploration of pioglitazone’s effects in lung cancer chemoprevention or treatment warrants a large cohort study to support precise conclusions, clinical trials in relapsed or refractory (r/r) melanoma, renal clear cell carcinoma (RCCC), Hodgkin’s lymphoma (HL), multisystem Langerhans cell histiocytosis (mLCH), and non-promyelocytic acute myeloid leukemia (non-PML AML) demonstrated the induction of tumor cell death as documented by complete remission (CR) in r/r non-PML AML, continuous CR in r/r RCCC, and mLCH [90]. In HL patients, the CR was observed by the combination of pioglitazone and everolimus. These findings suggest tumor-specific biomodulatory efficacy of pioglitazone across various histologic neoplasias. Nevertheless, the question whether pioglitazone uniquely modulates NSCLC pathways is still debatable given its potent anticancer efficacy in other cancer models, which is attributed to its ability to target multiple signaling pathways and induce pro-apoptotic effects either alone or in combination with other therapeutic agents [91,92,93,94].

## 4. Conclusions and Future Perspective

Pioglitazone, a known PPARγ agonist, has shown encouraging potential as a therapeutic agent for NSCLC. Extensive in vitro and in vivo studies supported its ability to reduce tumor growth, induce apoptosis, limit invasiveness, and attenuate angiogenesis. Mechanistically, pioglitazone targets the key signaling pathways and cellular activities such as MAPK, NF-κB, TGF-β/SMAD, and EMT, which play vital roles in cancer progression. These diverse mechanisms suggest that pioglitazone can influence several cancer-related processes at once, giving it an advantage as a multi-targeted therapy. However, translating these findings into clinical benefit has been challenging. For example, a Phase II trial by Keith and colleagues confirmed the drug’s safety but did not show any significant histological improvement in high-risk smokers. Rather than being a setback, this highlights the complexity of lung cancer chemoprevention and points to areas where further work is needed—such as better patient selection, longer treatment periods, or combination strategies.

Moving forward, the future of pioglitazone in NSCLC will likely depend on more precise and personalized approaches. To that end, research should focus on identifying biomarkers that predict treatment responses, such as EGFR gene mutation (e.g., T790M and L858R), that confer improved outcomes such as significantly longer progression-free survival in advanced NSCLC patients treated with EGFR-TKIs (e.g., osimertinib), with or without a combination of chemotherapy ([95,96,97]). Such biomarkers could be explored in diabetic NSCLC patients with a combination of PPARγ agonists in future studies.

Another potential strategy involves exploring newer/selective PPARγ modulators that may provide improved safety or efficacy profiles. For example, the novel PPARγ ligand CB13 exerts potent anticancer effects by activating PPARγ, which in turn promotes the generation of ROS and induces endoplasmic reticulum (ER) stress. The combined actions of ROS and ER stress lead to apoptosis through inhibition of the PKR-like endoplasmic reticulum kinase (PERK)–eukaryotic translation initiation factor 2 alpha (eIF2α)–activating transcription factor 4 (ATF4)–C/EBP homologous protein (CHOP) pathway. Through these mechanisms, CB13 decreases cell viability, enhances cytotoxicity, and promotes programmed cell death in NSCLC cells. Notably, CB13 also sensitizes radioresistant NSCLC cells to radiation, thereby enhancing their therapeutic susceptibility [98].

However, meta-analyses of other hypoglycemic drugs have reported mixed results. Govindarajan and colleagues conducted a retrospective study analyzing data from 87,678 diabetic male patients aged ≥40 years treated at 10 Veteran Affairs medical centers between 1997 and 2003 to evaluate whether TZDs influence cancer risk. Patient records were reviewed for subsequent diagnoses of colorectal, lung, and prostate cancer, and statistical models were adjusted for potential confounders such as age, race, body mass index, and use of insulin or other antidiabetic drugs. The results showed that TZD use was associated with a 33% reduction in lung cancer risk (relative risk 0.67; 95% CI, 0.51–0.87), whereas the reductions in colorectal and prostate cancer risk did not reach statistical significance. The study concluded that TZD exposure may confer a protective effect against lung cancer while emphasizing the need for further studies to confirm and elucidate these findings [99].

Along similar lines, Wu and colleagues performed a meta-analysis of 15 studies (11 cohort studies, 2 case–control studies, and 2 randomized control trials [RCTs]) to determine the association between conventional glucose-lowering drugs and lung cancer risk [100]. The results showed that metformin use was associated with a 15% reduction in lung cancer risk in observational studies (OR 0.85, 95% CI 0.77 to 0.92); however, this protective effect was not observed in analyses adjusted for smoking or restricted to RCTs. TZDs and sulfonylureas showed no significant effect on lung cancer risk (OR, 1.10; 95% CI, 0.93 to 1.26 and OR, 0.86; 95% CI, 0.70 to 1.02, respectively), while insulin use was linked to a 22–23% increased risk (OR 1.23, 95% CI, 1.10 to 1.35), an association that persisted even after adjusting for smoking. The study concluded that metformin may exert a protective effect, insulin use may increase the risk of lung cancer, and TZDs and sulfonylureas appear to have no significant impact. As most of the evidence was derived from observational data, the study emphasized the need for large, well-controlled randomized trials to confirm the associations between glucose-lowering drugs and lung cancer risk [100].

Overall, these findings suggest that with continued investigation, pioglitazone may have the potential to extend beyond its traditional use as an antidiabetic drug and take on a more prominent role as a promising candidate in the management of NSCLC.

## Data Availability

No new data were created or analyzed in this study.

## Abbreviations

NSCLC	Non-small-cell lung cancer
PPARγ	Peroxisome proliferator-activated receptor-gamma
MAPK	Mitogen-activated protein kinase
EMT	Epithelial-to-mesenchymal transition
NR1C3	Nuclear receptor superfamily 1 group C member 3
NADPH	Nicotinamide adenine dinucleotide phosphate
ALDHs	Aldehyde dehydrogenase
ROS	Reactive oxygen species
POX	Proline oxidase
PTEN	Phosphatase and tensin homolog
ERK	Extracellular signal-regulated kinase
GADD153	Growth Arrest and DNA Damage
